# Outcome of Very Premature Newborn Receiving an Early Second Dose of Surfactant for Persistent Respiratory Distress Syndrome

**DOI:** 10.3389/fped.2021.663697

**Published:** 2021-04-30

**Authors:** Eva Greiner, Apolline Wittwer, Eliane Albuisson, Jean-Michel Hascoët

**Affiliations:** ^1^Department of Neonatology, CHRU, Nancy, France; ^2^CHRU-Nancy, Direction de la Recherche et de l'Innovation (DRI), Département MPI, Unité de Méthodologie, Data management et Statistique (UMDS), Nancy, France; ^3^Lorraine University, Vandoeuvre les Nancy, Nancy, France

**Keywords:** premature, two doses surfactant, neonatal respiratory distress syndrome, outcome, mortality

## Abstract

**Background:** Infants presenting respiratory distress syndrome (RDS) not responding to surfactant often receive a second instillation. Few studies evaluated the consequences of this second administration. This study aimed at determining the outcome of infants presenting persistent RDS and receiving an early second dose of surfactant.

**Methods:** Infants below 32 weeks' gestation who received a second dose of 100mg/kg of surfactant within the first 72 h of life, were retrospectively involved in this 42 months' study. They were matched to two controls receiving a single dose of 200mg/Kg based upon gender and gestational age.

**Results:** 52/156 infants receiving two doses (Group 2-doses) were significantly more often SGA [22 (42%) vs. 21 (20%) *p* = 0.04] and outborn [29 (56%) vs. 13 (12%) *p* = 0.001]. They had received antenatal corticos teroid therapy less often [26 (50%) vs. 89 (86%) *p* = 0.001] and presented more severe RDS based upon FiO2 level, oxygenation index and radiography. Group 2-doses survival was lower (65.4% vs. 79.6 % *p* < 0.1) but surviving infants did not have different morbidity than controls.

**Discussion:** Premature newborn receiving a second dose of surfactant had adverse antenatal characteristics, presented more severe RDS and only partially responded to the first dose. Outcomes of surviving infants who received 2 doses of surfactant were comparable to others.

## Keynotes

- Newborn receiving a second dose of surfactant had adverse antenatal characteristics, presenting with more severe respiratory distress syndrome initially and only partially responded to the first dose.
- Infants mortality was higher when they had received 2 doses of surfactant.
- Outcomes of surviving infants who received 2 doses of surfactant were comparable to others.

## Introduction

Respiratory distress syndrome (RDS) is a progressive respiratory failure caused primarily by a deficit of pulmonary surfactant and a structural immaturity of preterm infants' lungs. It is a major cause of morbidity and chronic lung disease. According to the latest European recommendations considering the generalization of antenatal corticosteroids (1), prophylactic administration of surfactant in preterm infants is no longer a valid option. Early administration of surfactant is now recommended for preterm infants who need intubation and for very preterm infants whose mothers did not receive antenatal corticosteroids. A fraction of inspired oxygen (FiO2) of 30% or more after 2 h of continuous positive airway pressure (CPAP) may be predictive of CPAP failure and should indicate surfactant replacement therapy (2).

More recent approaches to deliver surfactant while minimizing invasive ventilation have been proposed (3): less invasive surfactant administration (LISA) (2) or similar procedure [MIST: minimally invasive surfactant therapy (3)], using an intra tracheal catheter or a feeding tube during CPAP without intubation and mechanical ventilation. However, in case of persistent RDS after a first dose of surfactant, the management remains controversial and the use of a second dose of surfactant is a common but poorly defined practice in timing and dosing. A second dose of surfactant has been suggested as an option when encountering difficulties in weaning from mechanical ventilation, with chest X-rays showing persistence of RDS few hours after the first dose. Studies have used an ultrasound score to better identify the need for a second dose of surfactant (4–6).

In 1993, Halliday et al. (7) compared high and low dose surfactant regimen for the treatment of RDS and showed that the high dose regimen (total dose of 600 mg/kg) of Poractant alfa was not superior to the low dose one (total dose of 300 mg/kg). On the other hand, according to Dunn et al. in1990 (8), the administration of several doses of Beractant seemed more effective, but the optimal dosage and time interval between doses were not clearly defined. Of note, this study was carried out on more mature newborn (30–36 weeks of gestation). In a Cochrane meta-analysis, Soll et al. (9) demonstrated that a multiple dose regimen of surfactant, rather than a single dose, further reduced the risk of pneumothorax (RR 0.51, 95% CI 0.3–0.88) and was associated with a tendency toward a reduction in mortality. Another study by Speer et al. (10) showed a decrease in mortality and pneumothorax in neonates receiving 3 doses (400 mg/kg) of Poractant alfa.

All above studies, conducted at a time when antenatal corticosteroids were not yet routinely used, showed decreased rates of pneumothorax and a tendency toward a reduction in mortality in children who received more than one dose of surfactant for persistent RDS after the first dose.

Figueras Aloy et al. (11) worked on defining an optimal timing of the administration of the second dose of surfactant. The 57 newborn included in their study received a second dose of surfactant at either 2 or 6 h of life. Results showed a moderate improvement in premature infants of <1,000 g having received the second dose at 2 h of life.

The official recommendation guidelines state that “an additional dose of 100 mg/kg may be administered 6 to 12 h after the first dose to neonates with persistent signs of respiratory distress and remaining on ventilatory support. The cumulative total dose should not exceed 400 mg/kg. ”(12).

Few studies on the administration of two doses of surfactant are available and do not define a target population. We still do not really know whether a second dose of surfactant instillation is indeed beneficial. Many questions remain unanswered regarding the time delay between the first and second dose, the correct dosage to be administered and the criteria required for its administration. The aim of our study was to determine primarily the survival then secondary the short-term outcomes of very premature newborn presenting with persistent RDS who received a second dose of exogenous surfactant. Other secondary outcomes were to characterize this population of premature newborn and compare it to the infants who only received one dose of surfactant over the same period of time, in order to determine criteria to select which newborn could benefit from the administration of a second dose of surfactant.

## Materials and Methods

Very premature infants were involved in a monocentric retrospective cohort study in the level 3 maternity hospital of Nancy from November 1st, 2013 to April 30th, 2017 using infants' medical records. All infants born before 32 weeks of gestation and who received two doses of surfactant were included in the study. This study was approved by the Commission Nationale Informatique et Libertés and recorded under the number R2018-09.

Infants who presented congenital heart disease or major congenital malformations were excluded.

The primary outcome measure was survival. Secondary outcome was short-term morbidity of preterm neonates who received a second dose of surfactant for persistent RDS after a first dose of 200 mg/Kg of Poractant alfa. A favourable evolution was defined by survival and a length of hospitalization below the 3rd quartile of the hospitalisation duration of newborns receiving a single dose of surfactant.

Perinatal data included obstetric and neonatal information: gestational age (GA), calculated with date of last menstruation and first trimester ultrasound; birth weight; gender; single or multiple pregnancy; mode of delivery; antenatal corticosteroids; small for gestational age (SGA) defined by a birth weight less than the 10th centile of the weight expected for their gestational age, evaluated by Fenton growth chart (13); premature rupture of membranes (PRM); 1-min and 5-min Apgar score; umbilical cord pH; early onset sepsis; ventilator settings; timing and dosage of the first and second dose of surfactant; severe RDS was defined by a grade greater than or equal to grade 3 radiologically.

We also recorded all additional respiratory treatments: Doxapram, caffeine, inhaled NO, and corticosteroids.

Then, we evaluated morbidity and mortality (events and dates) by the incidence of death, patent ductus arteriosus (PDA) requiring treatment, necrotizing enterocolitis (NEC) as defined by Bell (14), retinopathy of prematurity (ROP) stage 2, 3 and higher (15), late onset sepsis, pneumothorax, severe intraventricular haemorrhage defined by a grade of 3 or 4 (16), duration of mechanical ventilation and bronchopulmonary dysplasia (BPD). BPD was defined as mild when the infants required 21% of FiO2 at 36 weeks of gestation, as moderate when they required oxygen with FiO2 < 30%, and severe when they required oxygen with FiO2 ≥ 30%. (17).

### Statistical Analysis

Fifty-two patients received 2 doses in the cohort of 400 very premature infants born during the study period. Two controls per patient, paired for sex and gestational age, were chosen for the analysis. Thus, 156 patients were evaluated in this study.

Normally distributed data are presented as mean values with SD; non-normally distributed data are presented as medians with range or Inter Quartile Range (IQR). A Chi2 test or Fisher exact test was used when appropriate for categorical variables. For continuous variables not normally distributed, we used the Mann-Whitney *U*-test in comparisons.

As the FiO2 values after the first dose of surfactant have non-normal distributions and as our aim was more to identify subgroups of subjects than to establish an equation for the whole sample, we did not use multivariate regression models because of the instability that would have affected some coefficients. We chose a strategy allowing us to hierarchize variables and identify subgroups of subjects with respect to the SGA, Maturation, Outborn and FiO2 1h after the first dose of surfactant. We used a Chi-squared automatic interaction detector (CHAID) method. The IBM SPSS statistic software has extended CHAID algorithms to handle target or predictor variables even if they are categorical, ordinal or continuous. An alpha level of 0.05 was chosen as a significant difference. All analyses were performed with SPSS IBM Statistics V25.

## Results

From November 1st, 2013 to April 30th, 2017, 52 out of 400 newborns born before 32 weeks of gestation (13%) receiving early surfactant eventually had two instillations within the first 72 h of life (Group 2-doses). Gestational age was 27.0 ± 1.9 (Mean ± SD); range (24–31.6) WGA and birth weight 972 ± 337g. These children were matched on sex and term of birth with 104 children who received a single dose of surfactant over the same period (Group 1-dose).

Newborns' adaptation to extra uterine life, evaluated on cord pH value (*p* = 0.18) and 1-min and 5-min Apgar score (*p* = 0.15; *p* = 0.16), was not significantly different between the two groups (Table 1).

**Table 1:** Perinatal characteristics of the neonates.

	**Group 1-dose** **(104)**	**Group 2-doses** **(52)**	**p**
Males, *n* (%)	54 (52)	28 (54)	0.82
Gestational age, mean, (sd, range), weeks	27.4 (1.9, 24–31.7)	27.2 (2.0, 24–31.6)	0.44
Birth weight, mean (sd),	982.5 (338.1)	953.8 (336.1)	0.46
SGA, *n* (%)	**21 (20)**	**22 (42)**	**0.004**
Prenatal corticosteroids, *n* (%)	**89 (86)**	**26 (50)**	**0.001**
Incomplete steroid maturation, *n* (%)	**12 (13)**	**11 (48)**	**0.001**
Outborn, *n* (%)	**13 (12)**	**29 (56)**	**0.001**
Caesarean section, *n* (%)	54 (52)	28 (53)	0.82
Premature rupture of membranes, *n* (%)	32 (30)	21 (40)	0.23
Multiple pregnancy, *n* (%)	23 (22)	19 (37)	0.05
Mfi *n* (%)	15 (28)	30 (29)	0.97
Apgar score (median, IQR:1/5 min)	3(2,6)/6(5,7)	3(1,5)/6(5,7)	0.15/0.16
pH at birth, mean (sd)	7.30 (0.10)	7.26 (0.15)	0.18

*SGA, small for gestational age; pH, potential hydrogen; MFI, maternal-foetal infections. Bold values are statistically significant*.

In Group 2-doses, infants were significantly more often SGA [22 (42%) vs. 21 (20%) (*p* = 0.04)] and outborn [29 (56%) vs. 13 (12%) (*p* = 0.001)] than in Group 1-dose. They had received antenatal corticosteroids therapy less often [26 (50%) vs. 89 (86%) (*p* = 0.001)] and often incompletely [11 (48%) vs. 12 (13%) (*p* = 0.001)] (Table 1). The mean time of administration of the second dose of surfactant (100 mg/kg dosing) was 15 h 30 min ± 42 min of life; range (3-64) h.

### Primary Outcome

**Figure 1:**
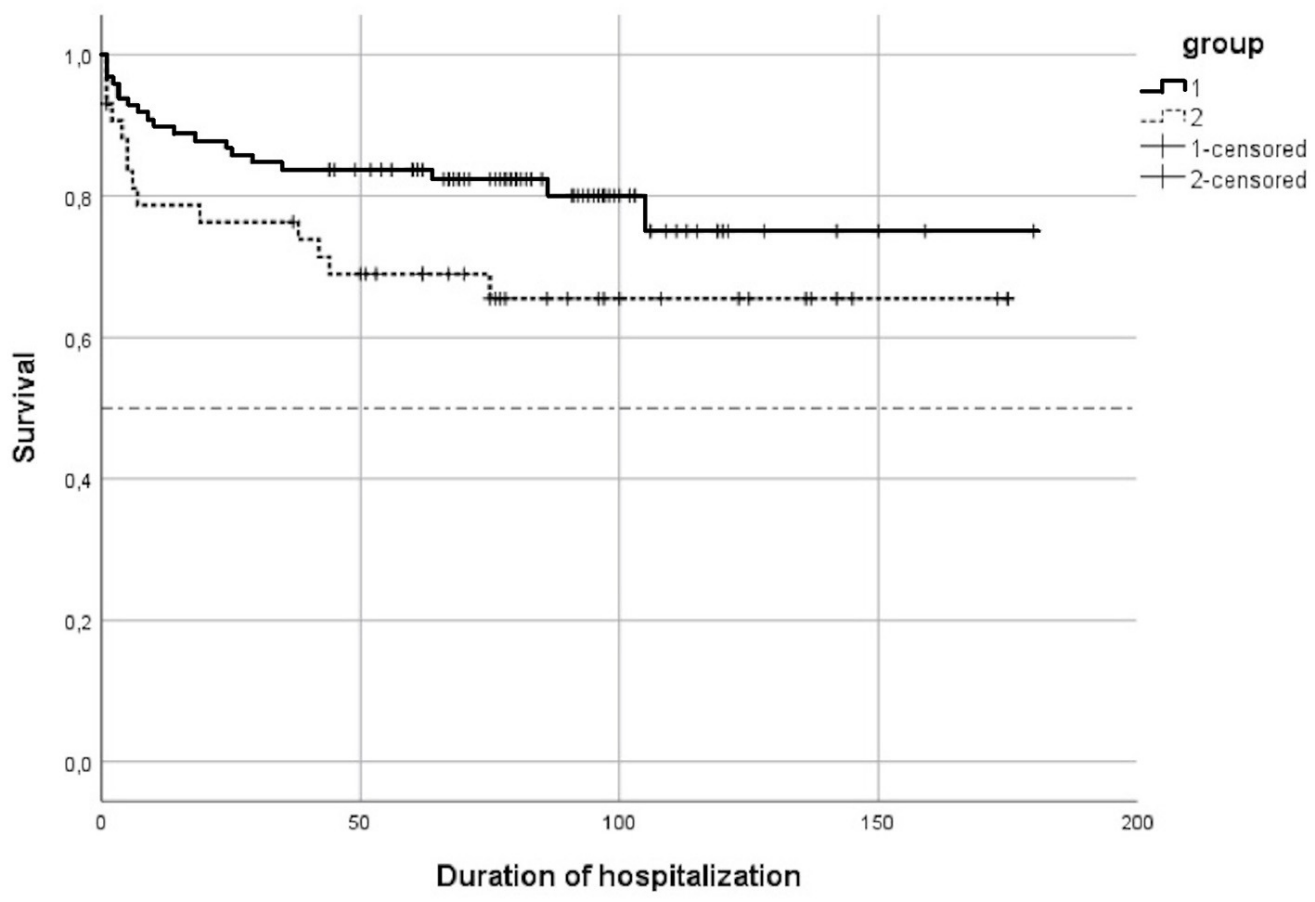
Survival curve.

The survival was lower in Group 2-doses (65.4 vs. 79.6 %, *p* = 0.049) (Figure 1).

Median duration of hospitalization for the surviving infants in Group 1-dose was 77.5 days with an interquartile range (IQR) = (59; 97). In Group 2-doses, the median duration of hospitalization was 67 days, and 20 surviving infants (58, 9 %) had a duration of hospitalization lower than or equal to 97 days, the 3rd quartile of Group 1-dose duration of hospitalisation.

For these infants with a favourable evolution, mean gestation age was 28.7 (25.2–31.0) weeks of gestation, mean birth weight was 1224g (880-1610g), SGA was present for 6 of them, and half of them were outborn. There were 7 multiple pregnancies and 8 children received antenatal corticosteroids, including 4 partial courses.

### Secondary Outcomes

#### Respiratory Status Before Surfactant Administration

A significant difference was found both for the oxygenation index (Paw x Fio2/PaO2), (*p* = 0.008) and Fi02 (*p* = 0.001) which were higher in Group 2-doses (Table 2). Newborn infants in Group 2-doses had significantly more severe HMD. Surfactant was administered earlier in Group 2-doses (1 h after birth *vs*. an hour and a half in Group 1-dose, *p* = 0.02) (Table 2).

**Table 2:** Respiratory criteria before surfactant administration.

	**Group 1-dose** **(104)**	**Group 2-doses** **(52)**	***P***
Severe hyaline membrane disease, *n* (%)	**42 (41)**	**43 (84)**	**0.001**
Fi02	**0.5 (103) [0.3; 0.7]**	**0.7 (50) [0.59; 0.8]**	**0.001**
Time of the first dose (hour since birth)	**1.5 (103) [1;2]**	**1 (51) [1;2]**	**0.02**
Oxygenation index	**8.28 (73) [3.8; 13.5]**	**10.6 (34) [7.9; 13.8]**	**0.008**
Respiratory rate (cycles/min)	48.5 (102) [40; 50]	50 (47) [45; 55]	0.10
Pep in cm h 20	4 (99) [4; 4]	4 (47) [4; 4]	1
tcPCO2 in mmhg	51 (91) [44; 56]	51 (35) [44: 60]	0.58
Conventional mechanical ventilation, *n* (%)	99 (96)	46 (92)	0.28
Dosage of the first dose mean (mg/kg)	200 (103) [200; 200]	200 (52) [200;200]	1
Airway pressure in cm h 20, median (*n*) [IQR]	8.2 (74) [7.7; 9]	8.3 (39) [7.5; 9.1]	0.63

*PEP, positive expiratory pressure; tcPCO2, transcutaneous pressure in CO2; PAW, airway pressure; PaO2, pressure in O2. Bold values are statistically significant*.

#### Respiratory Outcome After the First Dose of Surfactant

After the first dose of surfactant, FiO2 decreased significantly in both groups, with a more important decrease in the two-doses group who started with a significant higher FiO2 level (0.23 vs. 0.31, *p* = 0.001). Oxygenation index also decreased more in Group 2-doses (3.28 vs. 5.20, *p* = 0.001). There was no statistically significant difference in airway pressure (*p* = 0.66) and positive expiratory pressure (*p* = 0.98) between the two groups (Table 3).

**Table 3:** Respiratory criteria after surfactant administration.

	**Group 1-dose** **(104)**	**Group 2-Doses** **(52)**	***p***
Fi02	**0.23 (102) [0.21;0.29]**	**0.31(48)[0.26;0.53]**	**0.001**
Respiratory rate (cycles/min)	**40 (101) [30; 50]**	**50 (46) [40; 55]**	**0.001**
tcPCO2 in mmhg	**43 (94) [38.8; 49]**	**47 (39) [43: 59]**	**0.001**
Oxygenation index	**3.28 (79) [2.5; 4.5]**	**5.2 (35) [3.3; 7.4]**	**0.001**
Pep in cmh 20	4 (90) [3; 4]	4 (42) [4; 4]	0.98
Conventional mechanical ventilation, *n* (%)	90 (87)	43 (90)	0.69
Airway pressure in cm h 20, median (n) [IQR]	8.0 (80) [7.0; 9.1]	8.1 (40) [7.4; 9.0]	0.66

*PEP, positive expiratory pressure; tcPCO2, transcutaneous pressure in CO2; PAW, airway pressure; PaO2, pressure in O2. Bold values are statistically significant*.

Infants receiving two doses of surfactant had significant higher transcutaneous PCO2 [43 vs. 47 mmHg (*p* = 0.01)] and respiratory rate [40 in Group 1-dose vs. 50 in Group 2-doses (*p* = 0.001)] (Table 3).

**Figure 2:**
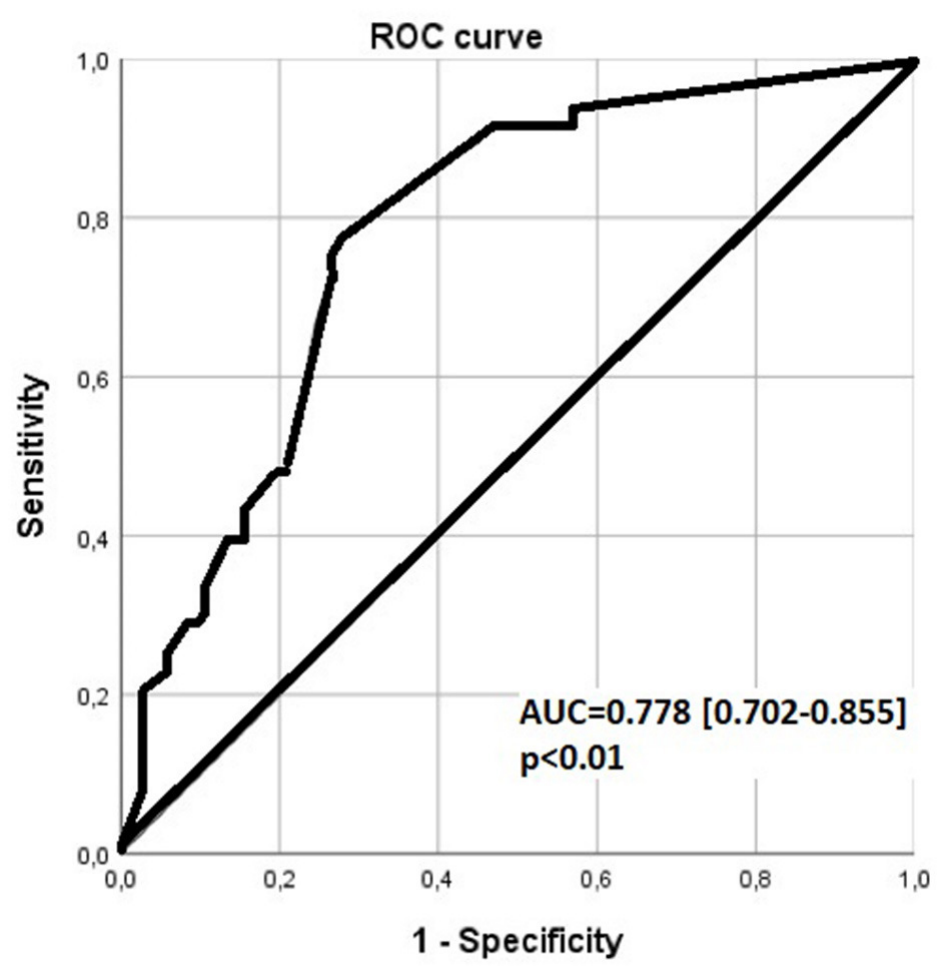
FiO2 level for a second instillation of Surfactant.

FiO2 level > 0.3 1 h after the first dose of surfactant, was associated with a sensitivity of 0.67 and a specificity of 0.76 for receiving a second dose, by ROC curve analysis with an area under the curve (AUC) (0.95 CI) = 0.778 (0.702–0.855) (Figure 2).

#### Morbidity

Bronchopulmonary dysplasia rates were not different between the two groups (*p* = 0.88). Neither were other neonatal morbidities such as late-onset sepsis, cerebral intraventricular haemorrhage or ventricular dilatation, necrotising enterocolitis, and retinopathy of prematurity (Table 4).

**Table 4:** Neonatal outcome.

	**Group 1-dose (104)**	**Group 2-Doses (52)**	***p***
Sepsis, *n* (%)	59 (57)	29 (55)	0.88
Severe bpd, *n* (%)	39 (38)	19 (37)	0.87
Bpd, *n* (%)	40 (39)	12 (25)	0.09
Patents ductus arteriosus, *n* (%)	42 (40)	29 (58)	0.04
Severe intraventricular hemorrhage, *n* (%)	10 (9)	7 (13)	0.59
Rop, *n* (%)	25 (24)	18 (36)	0.36
Nec, *n* (%)	10 (10)	6 (11)	0.78
Doxapram treatment, *n* (%),	53 (51)	23 (46)	0.56
Caffeine treatment, *n* (%)	90 (86)	39 (76)	0.12
Corticosteroid treatment, *n* (%)	42 (40)	23 (46)	0.51
Pneumothorax, *n* (%)	2 (2)	4 (8)	0.09
Mechanical ventilation duration (days), med (n) [Q1; Q3]	5 (104) [1;11.8]	6 (52) [4;15.7]	0.03
O2 duration (days), med (*n*) [Q1; Q3]	52.5 (104) [29.8;76]	41.5 (48) [7.3; 74.8]	0.31
Hospitalization duration (days), med (*n*) [Q1;Q3]	77.5 (98) [59;97]	67 (43) [19;100]	0.31
Hospitalization during the first year	1 (76) [0;2]	1 (27) [0;2]	1
Mortality, *n* (%)	21(20)	18(35)	0.05

*BPD, bronchopulmonary dysplasia; ROP, retinopathy of prematurity; NEC, necrotizing enterocolitis*.

Mean duration of mechanical ventilation was significantly lower in the one-dose group (5 days vs. 6 days, *p* = 0.03) (Table 4).

**Figure 3:**
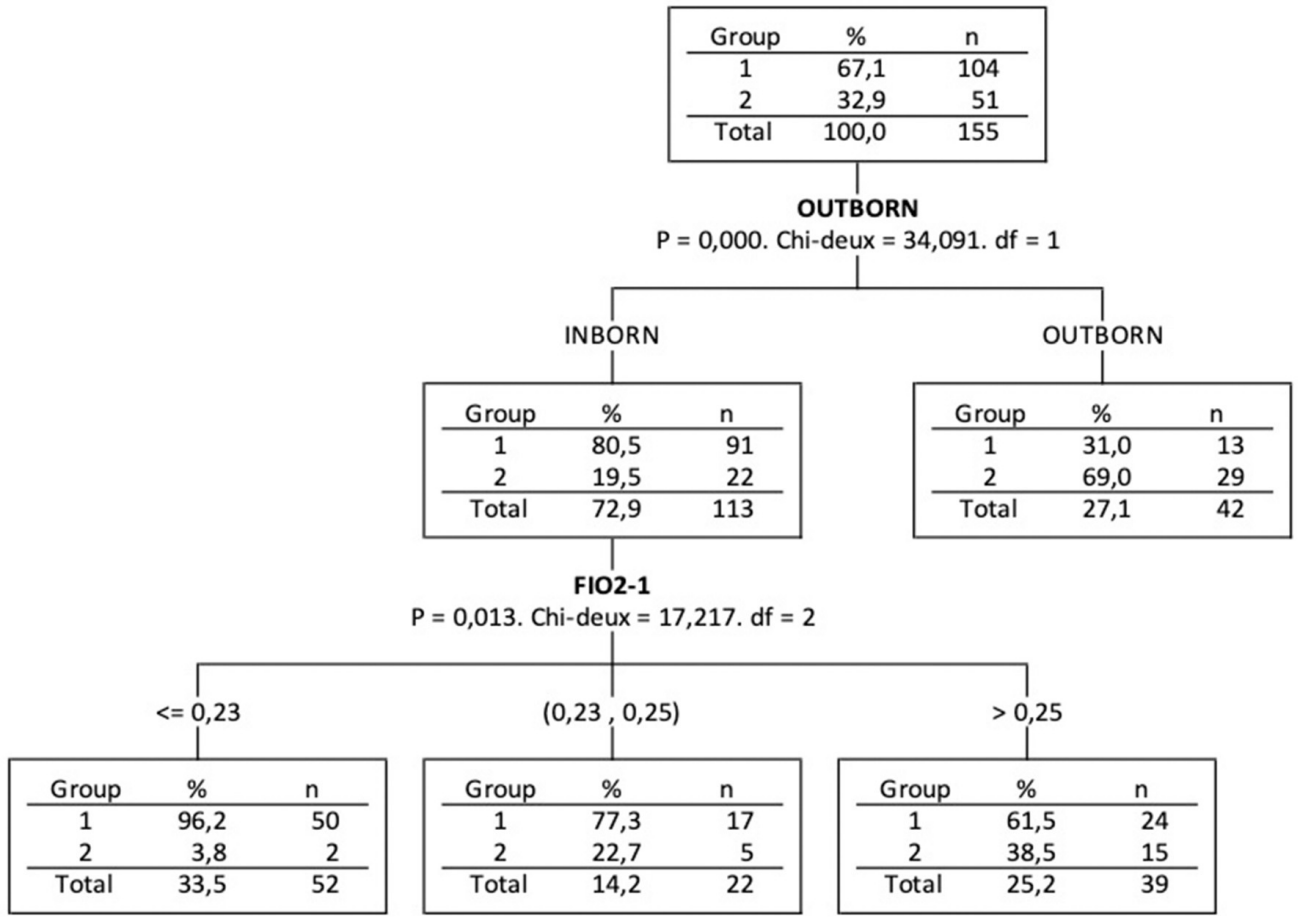
Decision Tree by CHAID method.

Based on CHAID decision tree, outborn (*p* = 0.0001) is the most discriminant variable among the 4 variables that were significant in bivariate analysis (Table 1: SGA, Maturation, Outborn and FiO2-1) analysed together to explain Group 2-doses association (Figure 3). For the inborn infants (outborn = 0), the second most discriminant variable is FiO2 1 h after first dose instillation (*p* = 0.013). We may notice that 57% of Group 2-doses infants were outborn and for the 23 inborn infants of Group 2-doses, a cut of FiO2-1 at about 0.23 concerned 91% of them.

#### Characteristics of the Infants Who Died

Table 5 shows the characteristics of the infants who died vs. those who survived in Group 2-doses. Infants who died also had incomplete corticosteroids maturation [12 (38%) vs. 11 (13%) infants *(p* = 0.003) respectively] and their adaptation to extra uterine life was worst in the group of dead infants as illustrated by significantly lower Apgar scores (Table 5).

**Table 5:** Perinatal characteristics of neonates comparing the dead and the living preterms in the group who received two doses of surfactant and comparing the dead in the two groups.

	**DEAD** **Group 2-doses (18)**	**DEAD** **Group 1-dose (21)**	**SURVIVING** **GROUP 2-Doses (34)**	***p_***a***_***	***p_***b***_***
Gestation age, mean, (range), WK	25.7 (24–28.8)	26.2(24.3–31.5)	27.8(24.3–31.5)	0.001	0.48
Birth weight, mean (range), g	785 (395–1,270)	820.1 (510–1,250)	1,043 (510–1,665)	0.01	0.69
Apgar score 1 and 5 min	3 [1-5]	3.5 [1-5]	4 [2–6]	0.032	0.28/0.19
Median, [IQR]	6 [2.3–7]	6.0 [4.5–7]	6 [5.5–7]	0.018	
Oxygenation index (paw x fio2 / pao2),mean (range)	16.2 (8.47–46)	12 (3.7–53)	12 (3.7–53)	0.037	0.18
Maturation by corticosteroids *n* (%)	11 (61)	3 (14)			0.03

*p_a_, comparison of dead in Group 2-doses vs. surviving in Group 2-doses*.

*p_b_, comparison of dead Group 2-doses vs. dead Group 1-dose*.

Respiratory settings were also significantly different, with a maximal FiO2 of 0.73 in the group of infants who died vs. 0.55 (*p* = 0,002), and an higher OI (Table 5). The timing of the first instillation of surfactant was not different between the infants who eventually died (14 h and 15 min of life) and the surviving infants (16 h and 30 min of life in average). Characteristics of dead infants in each group were compared and presented in Table 5. The only significant difference between the two groups was the absence of antenatal administration of corticosteroids for 44% in Group 2-doses vs. 14% in Group 1-dose (*p* = 0.03).

## Discussion

In this study we observed that the survival rate was significantly lower in the group receiving two doses of surfactant. This observation is in agreement with a recent study by Cosal et al. (18) who found a mortality of 26 vs. 35% in our study. Few publications are available with survival data for infants who received two doses of surfactant but, these infants were sicker with more severe RDS to start with. Thus, a poorer outcome was expected. The need for multiple doses of surfactant could potentially reflect severe underlying respiratory immaturity and associated gas exchange challenges in these extremely premature infants (12). In the study by Cosal and al (18) newborns had a lower GA and they may have received more than two doses of surfactant, while two was a maximum in our study.

The median length of stay for the 98 surviving infants who received a single dose of surfactant was 77.5 days with an upper quartile at 97 days. The median length of stay for infants who received two doses of surfactant was 67 days, 62% of them staying less than or 97 days. Therefore, we may consider that 62% of the infants receiving two doses of surfactant had a short term favourable outcome comparable to the infants who received one dose only. To the contrary of Cosal and al (18) we did not observe an increase in major morbidities such as ROP, pneumothorax, HIV or BDP in our population. This may be related to the lower GA in Cosal study suggesting that these morbidities are more related to the level of immaturity than to the instillation of a second dose of Surfactant when needed.

In our study, the evolution of the infants was similar in both groups in of terms of comorbidities and outcome. The duration of mechanical ventilation was one day longer in the group that received two doses which is not clinically significant with regards to the severity of the initial respiratory disease. Also, the duration of hospitalization was similar in both groups. So was the number of re-hospitalizations during the first year of life.

Over 42 months, 13% of preterm infants below 32 weeks of gestation (WGA) who had received a first early dose of surfactant had a second instillation within the first 72 h of life in our NICU. All patients were intubated shortly after birth. Early use of intubation may be explained both by the severity of the respiratory distress syndrome and by the large number of outborn infants whose airways needed to be secured for transportation to the level III NICU. Premature infants receiving a second dose of surfactant presented more antenatal criteria of bad prognosis and more severe RDS which is consistent with the study of Katz et al. (19).

In our study, infants who received a second dose of surfactant were significantly more often SGA or born from multiple pregnancies. They were more often outborn, received less antenatal corticosteroid therapy and when they did, it was more often incomplete. These results are consistent with the literature as a complete antenatal corticosteroid therapy would improve short and long term respiratory prognosis of the newborns (20).

As compared to infants who received only 1 dose of surfactant, infants in Group 2-doses did not differ significantly with respect to gender, method of delivery and the rate of premature rupture of membranes. Newborns' adaptation to extra uterine life, evaluated on the cord pH value and on 1-min and 5-min Apgar score, did not appear to differ between the two groups. These results suggest that perinatal anoxia was not associated to the administration of a second dose of surfactant. FiO2 requirements were significantly higher for the infants who eventually received a second dose of surfactant. But, the two groups did not differ significantly with respect to ventilator settings (respiratory rate, mean pressure, positive expiratory pressure) and tcPCO2. It would seem logical that infants with a more severe radiological respiratory disease and higher FiO2 requirements would also require more aggressive ventilation but it was not the case in our study.

All infants had received a first dose of 200 mg/kg of poractant alfa (Curosurf®). This recommended dosing is associated with a reduction of the need for a second dose of surfactant according to Singh and al (21). The timing of administration was slightly but significantly different between the two groups, Group 2-doses having received surfactant 15 min earlier in average. This difference may be explained by the frequent absence or incomplete antenatal corticosteroid therapy in Group 2-doses leading to a greater severity of RDS urging the use of surfactant. However, an impact of this difference of 15 min on the infant outcome is unlikely.

According to our study, a criterion that may indicate the need for administering a second dose of surfactant could be the oxygenation index which seemed to be a good indicator associated with RDS severity. Also, the oxygen requirements were significantly higher to start with (FiO2 of 0.7 vs. 0.5, respectively). Finally, RDS was significantly radiologically more severe in Group 2-doses. These three indicators appear to be appropriate to predict the severity of RDS and the need for a second dose of surfactant when the first dose is not rapidly successful. Indeed, there is also a difference regarding the response to the first dose of surfactant, despite it was administered with the same method and at the same dosage. One h after the administration of surfactant, FiO2 and OI were still significantly higher in the two doses group. So was the PCO2 level, despite a greater respiratory rate.

The mean delay of administration of a second dose of 100 mg/kg of surfactant was about 15 h and 30 min of life in average. In the literature there is no clear recommendation about the time of administration of the second dose of surfactant. Studies by Figueras et al. and Koskal et al. compared two groups who received a second dose 2 h vs. 6 h after the first one. They showed that the group with the shortest delay between the two doses presented greater respiratory improvement (11, 22). These results are consistent with our data suggesting that a shorter time could be recommended for the infants who are non-responders, as early as 1 h after the first dose in our study. The usual recommendation to give a second dose 6 to 12 h after the first one (12) is broad and may reflect the absence of appropriate data. To follow these recommendations, one could suggest considering 6 h as the very last limit after the first dose for indicating a second dose of surfactant, in case of persistent RDS.

In the studies allowing two doses, including ours, the response to the first dose of surfactant was significantly lower. The physiopathology of this lesser response remains unclear. Because infants needing a second dose had a significantly lower gestational age, less antenatal steroids, and more patent ductus arteriosus, lung immaturity leading to more vulnerability to barotrauma could be responsible of the lesser response to the first dose of surfactant. The study by Speer et al. describes a significant inflammatory process inducing lesions of the alveolar-capillary unit, allowing proteins of the serum to pass into the alveolar lumen and decreasing the effectiveness of the surfactant. An early repeated dose of surfactant might then help to reduce and prevent somehow this inflammation (23). One of the limitations of our study is the fact that it was a retrospective study. Because the prevalence of a second dose of surfactant is rather low (13%) the power of the study is low despite this was a retrospective study of all cohort of patients. Anyhow, we did not observe any significant difference for morbidity in the survivors. Thus, our study suggests that giving a second dose of surfactant seems indeed rather relevant. Another limitation is the large number of intubated children, the intubation criteria in our units should be re-evaluated.

## Conclusions

The administration of a second dose of surfactant in very preterm infants is difficult to predict. Nevertheless, our study showed that outborn birth was the main indicator of the need for a second dose. Physicians should also be alert in case of the absence of appropriate antenatal maturation, multiple pregnancy, and SGA. Some other indicators associated with an increased risk of a lesser response to a first dose of surfactant have been shown, such as a high oxygen requirement with an elevated oxygenation index and a high grade of severity on chest radiography or lung ultrasound. This observation could help to closely monitor these infants and administrate earlier a second dose of surfactant improving the overall outcome.

Thus, one could suggest that an FiO2 level > 0.3 1 h after a first dose of surfactant, associated with persistent signs of RDS, might be a guide to support a second dose of surfactant. Further studies are needed to determine more precisely the time of administration of a second dose of surfactant in case of persisting RDS after a first early dose.

## Data Availability Statement

The original contributions presented in the study are included in the article/supplementary material, further inquiries can be directed to the corresponding authors.

## Author Contributions

EG, AW, and J-MH contributed to conception and design of the study. EG organized the database and wrote the first draft of the manuscript. EA performed the statistical analysis. All authors contributed to the article and approved the submitted version.

## Conflict of Interest

The authors declare that the research was conducted in the absence of any commercial or financial relationships that could be construed as a potential conflict of interest.

## Abbreviations

BPD: bronchopulmonary dysplasia
CPAP: continuous positive airway pressure
FiO2: fraction of inspired oxygen
GA: Gestational age
HMD: hyaline membrane disease
Paw: airway pressure
Pep: positive expiratory pressure
PtCO2: transcutaneous pressure in CO2
RDS: Respiratory distress syndrome
PRM: premature rupture of membranes
SGA: small for gestational age.
